# Recent Progress of Three-Dimensional Graphene-Based Composites for Photocatalysis

**DOI:** 10.3390/gels10100626

**Published:** 2024-09-29

**Authors:** Fengling Zhang, Jianxing Liu, Liang Hu, Cean Guo

**Affiliations:** 1 School of Materials Science and Engineering, Shenyang Ligong University, Shenyang 110159, China; 2 School of Metallurgy, Northeastern University, Shenyang 110819, China; 3 School of Equipment Engineering, Shenyang Ligong University, Shenyang 110159, China

**Keywords:** graphene, aerogel, photocatalyst, application

## Abstract

Converting solar energy into fuels/chemicals through photochemical approaches holds significant promise for addressing global energy demands. Currently, semiconductor photocatalysis combined with redox techniques has been intensively researched in pollutant degradation and secondary energy generation owing to its dual advantages of oxidizability and reducibility; however, challenges remain, particularly with improving conversion efficiency. Since graphene’s initial introduction in 2004, three-dimensional (3D) graphene-based photocatalysts have garnered considerable attention due to their exceptional properties, such as their large specific surface area, abundant pore structure, diverse surface chemistry, adjustable band gap, and high electrical conductivity. Herein, this review provides an in-depth analysis of the commonly used photocatalysts based on 3D graphene, outlining their construction strategies and recent applications in photocatalytic degradation of organic pollutants, H_2_ evolution, and CO_2_ reduction. Additionally, the paper explores the multifaceted roles that 3D graphene plays in enhancing photocatalytic performance. By offering a comprehensive overview, we hope to highlight the potential of 3D graphene as an environmentally beneficial material and to inspire the development of more efficient, versatile graphene-based aerogel photocatalysts for future applications.

## 1. Introduction

Environmental pollution and energy deficiency restrict the sustainable development of human society. Solar energy is an inexhaustible clean resource, and the photocatalytic technology based on solar energy has the unique advantages of low energy consumption, sustainability, and the complete mineralization of pollutants without causing secondary contamination. As such, it is considered one of the most viable approaches for both environmental remediation and chemical energy production [1,2,3,4]. Photocatalysts are the core of photocatalytic technology, and since Fujishima’s [5] groundbreaking discovery of water photoelectrolysis using a semiconductor TiO_2_ electrode in 1972, there has been a concerted effort to develop more efficient photocatalysts and innovative strategies to enhance their performance. These advances have significantly propelled the field of photocatalytic technology. To date, numerous semiconductor materials have been reported in research pertaining to photocatalysis, including metal oxides, metal sulfides, vanadates, carbonitrides, and metal-organic frameworks (MOFs), among others (see Table 1).

**Figure 1:**
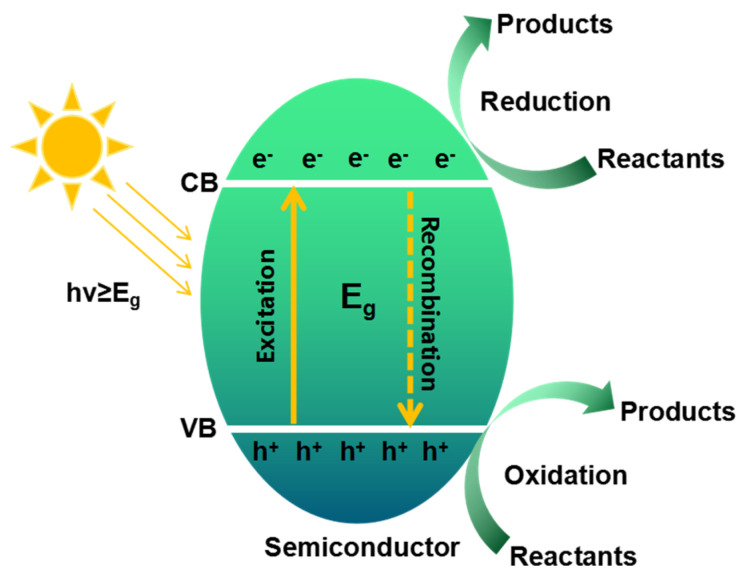
Scheme diagram of the photocatalytic process.

Photocatalytic reactions are complex physicochemical processes that typically unfold in several stages (Figure 1): (1) absorption of photons with energy greater than the semiconductor’s bandgap (Eg), leading to the generation of photoexcited electrons (e^−^) and hole (h^+^) pairs; (2) charge separation, followed by migration and partial recombination in the semiconductor particles; (3) redox reactions occurring on the surface of the photocatalyst [6,7,8]. It is now widely recognized that the effectiveness of photocatalytic reactions relies not only on thermodynamic constraints but also on kinetic conditions. The intricate charge dynamics and surface reaction kinetics are considered to be the key factors in determining the quantum yield of these reactions [9]. Nevertheless, single-component photocatalysts often face limitations, such as restricted light absorption and high rates of electron-hole recombination, which severely hinder their potential for large-scale industrial applications. In response, various design strategies have been proposed to fabricate photocatalysts with excellent functionalities through morphological control, noble metal deposition, ion doping, semiconductor coupling, and surface sensitization [10,11,12,13,14,15,16]. Developing photocatalysts with optimized structural properties and a deeper understanding of the relationship between structure and activity is therefore essential to advancing their functionality and broader applicability.

**Table 1:** Classification of photocatalysts.

Classification	Photocatalyst	Synthetic Method	Morphology	Application	Ref.
	TiO_2_	Anodic oxidation	Nanotube arrays	Degradation RhB and BPA	[17]
	ZnO	Calcination/Solvothermal	Nanorods	Degradation MB	[18]
	γ-Fe_2_O_3_	Precipitation/Calcination	Nanosheets	Oxygen evolution	[19]
Metal oxide	CuO	Ultrasound/Microwave	Flower-like	Cr (VI) reduction	[20]
	WO_3_	Precipitation/Calcination	Nanoflakes	Degradation CR and MB	[21]
	α-Bi_2_O_3_	Calcination	Nanoflowers	Degradation CV	[22]
	CeO_2_	Hydrothermal	Nanorods/Nanowires Octahedrons/Nanocubes	NO oxidation and CO_2_ conversion	[23]
	CdS	Etching/Sulfuration	Double-shelled nanocages	CO_2_ reduction	[24]
	MoS_2_	Hydrothermal	Flower-like	N_2_ fixation	[25]
Metal sulfide	FeS_2_	Hydrothermal	Nanorods	Degradation MB	[26]
	ZnS	Hydrothermal/Calcination	Nanosheets	H_2_ evolution	[27]
	CuS	Solvothermal	Microspheres	Degradation RhB, MB, MO	[28]
	Bi_2_S_3_	Solvothermal	Nanorods	N_2_ fixation	[29]
	BiVO_4_	Hydrothermal	Hollow nanotubes	Degradation CR	[30]
	FeVO_4_	Hydrothermal	3D nanowall-like	CO_2_ reduction	[31]
Vanadates	Ag_3_VO_4_	Hydrothermal	Mesoporous	Desulfurization	[32]
	InVO_4_	Microwave Hydrothermal	Nanocrystals Microspheres	H_2_ evolution	[33]
Carbonitride	g-C_3_N_4_	Molten salt	Nanorods	H_2_O_2_ generation	[34]
	C_3_N_5_	Molten salt	Nanosheets	H_2_ evolution	[35]
	Ti-MOF	Condensation	Needle-like	CO_2_ reduction	[36]
MOFs	Ni-MOF	Hydrothermal	Nanosheets	CO_2_ reduction	[37]
	Fe-MOF	Solvothermal	Hexagonal bipyramid	H_2_ evolution	[38]
	Co-MOF	Solvothermal	Nanosheets	Degradation dyes	[39]

Graphene is a two-dimensional (2D) planar carbon isomer with a honeycomb lattice structure formed by the sp^2^ hybridization of carbon atoms. Since its discovery by Geim in 2004, graphene has been recognized as a remarkable nanomaterial with exceptional optical transparency, electrical conductivity, mechanical strength, and chemical stability. Graphene provides a promising basis for photocatalytic applications due to its distinctive properties. There has been explosive interest in constructing various photocatalysts based on the versatile platform of graphene, ranging from simple semiconductor deposition to the precise control of multi-component growth, in the past few years [40,41,42,43,44,45,46]. Graphene-based nanocomposites with significantly enhanced photocatalytic performance have been widely employed in photochemical conversions, such as organic pollutants degradation, water splitting, CO_2_ reduction, N_2_ fixation, and photovoltaic application, etc. In photocatalytic reactions, the redox processes actually take place on the surface of the photocatalyst rather than in the liquid matrix. Effective photocatalysis depends not only on rapid charge separation and transfer but also on the ability of active sites on the photocatalyst surface to adsorb reactants [47,48]. Thus, designing photocatalysts with abundant active sites is critical for improving both the yield and selectivity of target products in target reactions. Graphene aerogel, as a derivative of graphene nanomaterials, is characterized by an orderly three-dimensional porous macroscopic structure combining mesopores and micropores, enabling the accommodation of plenty of nanoparticles on the framework or the skeleton due to its substantial surface area. Compared to its 2D counterpart, 3D graphene retains the beneficial properties of individual graphene sheets while avoiding the agglomeration caused by van der Waals interactions. Furthermore, its interconnected hierarchical porous structure provides easily accessible catalytic sites for reactive species, multidimensional electron transport pathways, and efficient channels for the mass transfer of electrolyte ions [49,50,51].

Over the past decade, some reviews have been conducted on 3D graphene-based materials, primarily focusing on the applications of graphene aerogels in energy storage (supercapacitors, batteries) [52,53,54,55], as well as their potential use as environmentally functional materials for pollutant removal [56,57,58]. However, comprehensive reviews specifically addressing the classification of 3D graphene-based photocatalysts and their diverse redox reactions remain relatively scarce. This review is dedicated to recent research breakthroughs in the synthesis and application of various types of 3D graphene-based photocatalysts and providing insights into the important role of 3D graphene in photocatalysis. We initially summarize the available synthetic methodologies for 3D graphene architectures. In the subsequent section, different types of 3D graphene-based photocatalysts are presented and their properties are highlighted. Subsequently, the application of these materials in photocatalysis is elaborated in detail, encompassing organic pollutant degradation, H_2_ evolution, and CO_2_ reduction. Finally, the challenge and prospective developments of 3D graphene in advanced multi-field photocatalytic applications are deliberated. It is expected that this paper may provide some support for the continuous advancement of the efficient design, manufacturing, and application of 3D graphene-based photocatalysts.

## 2. Basic Features

### 2.1. General Properties of Graphene and Its Derivatives

**Figure 2:**
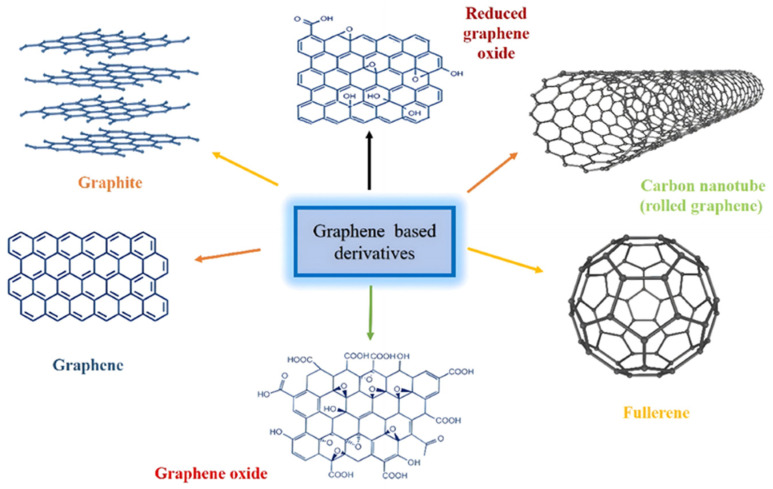
Systematic diagram of graphene and graphene-based derivatives. Reproduced with permission from Ref. [61], Copyright 2022, Elsevier.

Among carbon nanomaterials, graphene has emerged as a revolutionary material in both scientific and engineering research. Composed of a monolayer of carbon atoms arranged in a honeycomb lattice, graphene exhibits remarkable electrical and optical properties, including high electron mobility (~200,000 cm^2^ V^−1^ s^−1^), ballistic electronic transport, and outstanding transparency with an optical transmittance of approximately 97.7%. Additionally, graphene has a theoretical specific surface area of 2630 m^2^ g^−1^, excellent thermal conductivity (~5000 W m^−1^ K^−1^), strong chemical stability, and high mechanical strength [59,60]. A schematic illustration of graphene and its derivatives can be seen in Figure 2 [61]. Reduced graphene oxide (rGO) and graphene oxide (GO) constitute two commonly recognized forms of graphene derivative. GO is an oxidized derivative of graphene that contains numerous functional groups on its basal planes and edges, allowing it to form stable suspensions in various solvents and functioning as nucleation sites for the growth of other nanoparticles. However, GO is inherently less conductive than graphene due to the structural disorder induced by sp^3^ C-O bonds. GO can be reduced through chemical/thermal reduction or other methods to form rGO, enhancing its chemical stability and elevating its electrical conductivity [62]. As a highly versatile 2D building block, graphene has been assembled into 0D fullerenes, 1D carbon nanotubes, and 3D aerogels, all of which possess the potential to substantively extend the field of graphene applications. Notably, 3D graphene structures not only retain the inherent properties of graphene but also acquire new physicochemical characteristics. The 3D architectures constituted from 2D graphene sheets (including GO and rGO) via van der Waals forces or functional groups are easier to construct compared to other carbon materials [53]. Given these unique properties, 3D graphene, whether as a supporting material or synergistic component, already meets many of the essential requirements for advanced photocatalysts.

### 2.2. Functionalities of 3D Graphene for Photocatalysis

**Figure 3:**
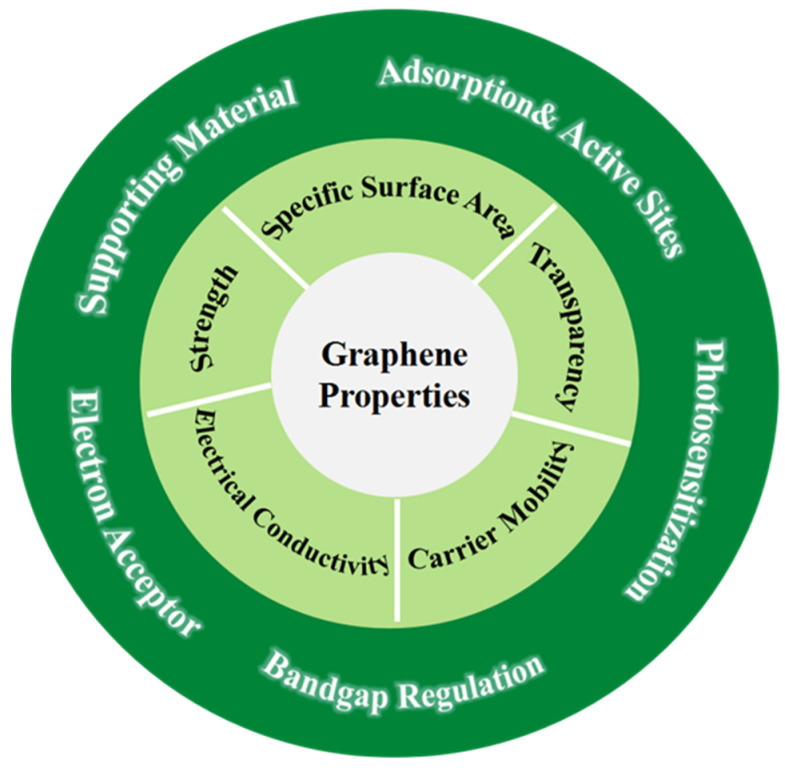
Various roles of 3D graphene in the photocatalytic system.

Optical and electrical properties are key factors in the development of photocatalytic materials. As research on graphene-based photocatalysts advances, it is important to highlight the role of 3D graphene within these systems. 3D graphene can improve the photocatalytic activity of composite photocatalysts in multiple manners, as schematically illustrated in Figure 3. **3D Graphene as a Supporting Material**; The large surface area and three-dimensional network of 3D graphene can support a substantial amount of semiconductor nanoparticles. Particularly, the negatively charged surface of reduced graphene oxide (rGO) facilitates the adsorption of nanoparticles, preventing agglomeration. This allows for controlled interfacial contact and the distribution of nanomaterials when precursors with varying compositions are added. Additionally, the diverse porous structure of 3D graphene (including micro-, meso-, and macropores) ensures better access for reactants and exposes more active sites, improving adsorption and surface photocatalytic reactions. **3D Graphene as a Photosensitizer**; In photochemical reactions, photosensitizers are capable of transmitting light energy to semiconductors that are not inherently sensitive to visible light, thereby enhancing or amplifying their photosensitive properties [63]. Graphene can function as both a metal with a vanishing Fermi surface and a semiconductor with a vanishing bandgap that stems from graphene’s honeycomb lattice composed of two equivalent carbon sublattices. This peculiarity can be regulated through the incorporation of heteroatoms or oxygen functionalities. The introduction of oxygen functionalities leads to the formation of C-O covalent bonds, disrupting the symmetry of the carbon sublattices and thereby altering their electronic properties. The bandgap is contingent upon the extent of graphene oxidation [64,65]. In regard to this, 3D graphene acquired through chemical redox methods can act as a photosensitizer, generating electrons, enhancing its light harvesting ability via multi-reflections within its structure, and modifying the bandgap of the photoactive components to expand the range of photoabsorption [66]. **3D Graphene as a Photoelectron Acceptor**; A major limitation of conventional photocatalytic materials is the rapid recombination of photogenerated electrons and holes, reducing the formation of energetic charge carriers. The efficient separation of these charges is critical for improving photocatalytic performance. Graphene’s electrical properties can be tuned by varying its oxidation level (C/O ratio), transforming it from a conductor to an insulator [67]. When electrically conductive 3D graphene is incorporated into photocatalysts, the formed three-dimensional conductive network offers rapid transport channels for photoelectrons, facilitating charge separation and promoting the overall photocatalytic efficiency. These multifunctional roles make 3D graphene a highly effective component in the design of advanced photocatalysts.

## 3. Synthetic Strategies of 3D Graphene Architectures

**Figure 4:**
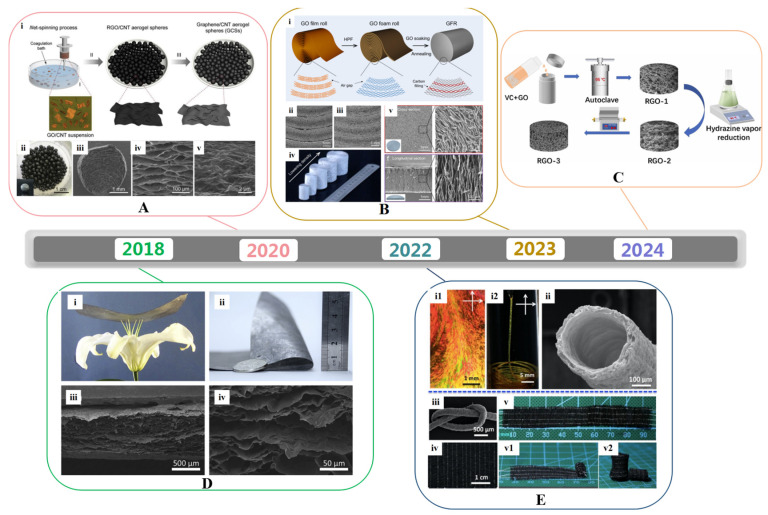
3D graphene with different structures. (**A**) Fabrication process (**i**) and morphology (**ii**–**v**) of graphene aerogel spheres. Reproduced with permission from Ref. [84], Copyright 2020, Elsevier; (**B**) Fabrication (**i**) and structural characteristics (**ii**–**v**) of graphene foam. Reproduced with permission from Ref. [85], Copyright 2023, Elsevier; (**C**) Schematic illustration for the preparation of graphene aerogel. Reproduced with permission from Ref. [86], Copyright 2024, Elsevier; (**D**) Digital camera images (**i**,**ii**) and cross-sectional view SEM images (**iii**,**iv**) of graphene aerogel film. Reproduced with permission from Ref. [87], Copyright 2018, Elsevier; (**E**) Cross polarized-light optical images (**i**), SEM images (**ii**,**iii**), and photographs (**iv**,**v**) of graphene aerogel hollow fiber. Reproduced with permission from Ref. [88], Copyright 2022, Elsevier.

Among the myriad 3D nanostructured materials, 3D graphene has garnered considerable attention owing to its diverse structural properties, encompassing a large surface area, low density, high porosity, and compressibility. Furthermore, 3D graphene is derived from 2D graphene by cross-linking together to form a network structure while retaining the exceptional electrical conductivity and physicochemical stability of graphene. These characteristics make it an efficacious scaffold for accommodating active materials across a wide range of applications in energy storage and conversion [68,69,70], photocatalysis [71,72,73], oil/water separation [74,75,76], electromagnetic shielding [77,78,79,80], sensors [81,82,83], and other fields. The choice of synthesis method plays a crucial role in customizing the morphology, size, defect structure, and surface/interface properties of 3D graphene materials. Numerous methods have been reported for fabricating 3D graphene and graphene-based structures, such as spheres [84], foam [85], aerogel [86], film [87], and hollow fiber [88], as shown in Figure 4. This review is dedicated to exploring the utilization of 3D structured graphene in photocatalysis; hence, the controllable preparation of well-ordered graphene macroscopic composites is a pivotal step. Additionally, attention should be paid to parameters such as size, specific surface area, porosity, bulk density, and surface wettability, which are affected by the preparation method. This section provides a brief overview of the main synthesis strategies for constructing 3D graphene architectures, which are typically divided into two categories: template and non-template methods.

### 3.1. Template Method

Template carbonization pioneered the utilization of SiO_2_ and NaCl as templates to precisely control both the morphology and pore structure of carbon materials during the 1980s and further extended this methodology to the construction of 1D, 2D, and 3D nanostructured carbon materials [89]. Diversifying template options significantly widened the synthetic possibilities for creating well-ordered 3D graphene materials. Essentially, alternative synthesis methods are based either on hard templates or soft templates. Among the hard templates, commonly used materials include metal foams (e.g., Ni, Cu), solid nanostructured particles (e.g., polystyrene spheres, silica, zeolites), and solidified solvents such as ice. These hard templates are instrumental in fabricating 3D graphene-based composites with varying macro- and microscopic structures. During the synthesis process, van der Waals forces along with ionic bonds play a pivotal role in assembling graphene components around the template. Once the template is removed, typically through etching or dissolution, a porous 3D graphene structure is left behind.

#### 3.1.1. Hard Template Method

##### Template-Directed CVD Method

**Figure 5:**
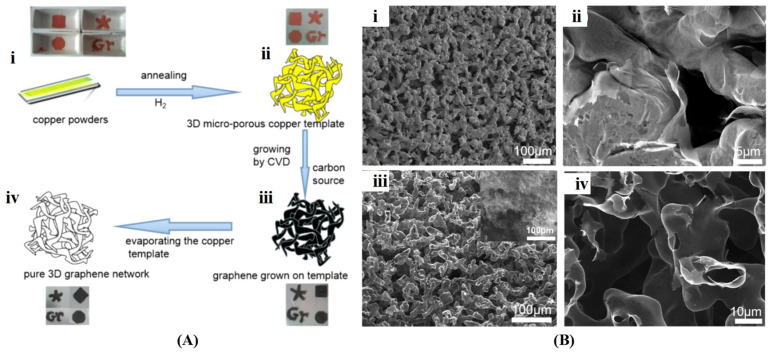
(**A**) Schematic diagram of the preparation process of 3D graphene by CVD (**i**–**iv**); (**B**) SEM images of copper template (**i**), graphene grown on copper template (**ii**), and graphene network after evaporating copper template (**iii**,**iv**). Reproduced with permission form Ref. [91], Copyright 2017, American Chemical Society.

Template-assisted chemical vapor deposition (CVD) is a highly effective method for the direct production of large-area and high-quality 3D graphene films by depositing carbon onto metal templates. The predominant architecture of the graphene is determined by the selection of different template forms and growth conditions, such as the flux and concentration of carbon sources. Graphene prepared using the CVD method closely resembles the physical structure of the original graphene sheet. Chen et al. [90] initially devised a methodology for the directional synthesis of 3D graphene through CVD by utilizing a nickel foam template. Graphene layers were formed on the surface of the template by the decomposition of CH_4_ at a temperature of 1000 °C. To maintain the integrity of the graphene network during the chemical etching process of the nickel template, a thin layer of polymethyl methacrylate was applied to support the graphene surface and subsequently removed by hot acetone. The resulting graphene retained the interconnected 3D scaffold structure of the nickel foam. Xia et al. [91] discovered that the stability of graphene samples is significantly affected by the removal of metal templates through solvent etching due to the introduction of defects and functional groups. To address this issue, they pioneered a solvent-free process using microporous copper as the template for creating a 3D graphene network. Following the growth of graphene via the CVD process using bubbling liquid ethanol as the carbon source, the copper template was evaporated at 1300 °C in a vacuum to obtain pure freestanding 3D graphene (Figure 5). Additionally, incorporating other precursors can lead to the formation of 3D graphene-based composites or doped structures. Jia et al. [92] fabricated 3D interconnected graphene foam/epoxy composites through a three-step process: (i) carbonization on a Ni foam template via CVD; (ii) impregnation of epoxy resin into the graphene-Ni foam followed by curing treatment; (iii) etching of the Ni template to create cellular-structured graphene/epoxy composites.

##### Microsphere Template-Assisted Method

**Figure 6:**
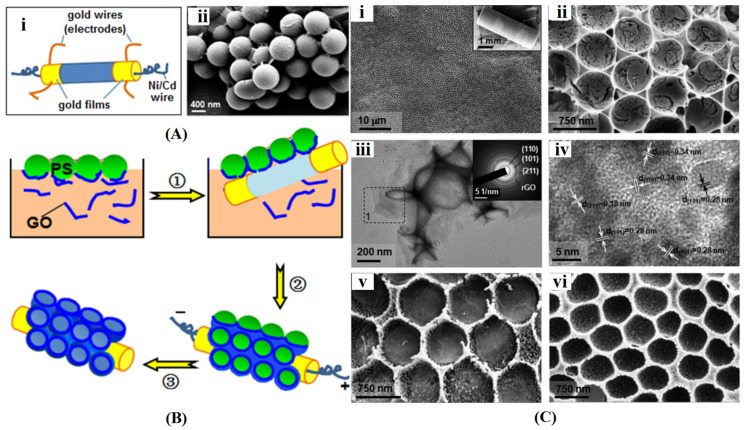
(**A**) Schematic of a ceramic tube (**i**) and SEM image of polystyrene microspheres wrapped with graphene oxide (**ii**); (**B**) Fabrication process of graphene MOP film; (**C**) Morphologies of graphene-oxide composite films: SEM images of graphene-SnO_2_ film (**i**,**ii**), TEM images of graphene-SnO_2_ film (**iii**,**iv**), and SEM images of graphene-Fe_2_O_3_ (**v**) and graphene-NiO (**vi**). Reproduced with permission from Ref. [94], Copyright 2016, American Chemical Society.

3D interconnected graphene frameworks created through the template method feature an organized microstructure, with their hierarchically porous architecture adjustable by modifying the size and concentration of template particles. Spherical polystyrene (PS) and silica particles are commonly used to develop high-surface-area graphene materials with micro-, meso-, and microporosity. Choi et al. [93] synthesized 3D graphene films with microporous structures using 2 μm PS spheres as templates. They first chemically reduced a graphene oxide suspension with ammonia and hydrazine to create a negatively charged graphene solution, which facilitated the uniform distribution of positively charged PS particles through electrostatic interactions. The mixed solution was subsequently filtered through an anodic membrane and peeled off to obtain composite films, and the final free-standing 3D graphene was achieved by removing the PS particles with toluene. Additionally, the prepared 3D graphene served as a scaffold for depositing a thin layer of MnO_2_ onto macropores via a self-limiting reaction to construct MnO_2_/graphene films. Xu et al. [94] utilized a PS microsphere monolayer colloid crystal as a template to fabricate ordered macroporous graphene-based films with tightly arranged pores on a curved surface (Figure 6). This fabrication process involved three steps: ① immersing the monolayer PS colloidal crystal, prepared by spin coating, in a graphene oxide solution; ② retrieving the colloidal crystals containing graphene oxide with a ceramic tube; and ③ heating the ceramic tube to 340 °C using a Ni/Cd alloy resistance wire to burn off the PS microspheres and reduce graphene oxide, resulting in macroporous graphene films. For further fabrication of graphene-incorporating (SnO_2_, Fe_2_O_3_, and NiO) composite film, the synthetic procedure remained consistent with that described above, except for directly dissolving specific materials into the graphene oxide solution. Salazar Aguilar et al. [95] employed a layer-by-layer method to construct 3D graphene by integrating spherical silica particles as templates. They first functionalized silica dispersions with a positively charged polyelectrolyte PEI to enhance interaction with the graphene oxide surface. The PEI-modified silica suspension was then added dropwise to the graphene oxide solution. After centrifugal washing of the silica particles covered with graphene oxide, the process was repeated with additional PEI modification and graphene oxide coating. Finally, thermal treatment and HF etching were employed for graphene oxide reduction and template removal to achieve the 3D graphene architecture.

##### Ice Template-Assisted Method

**Figure 7:**
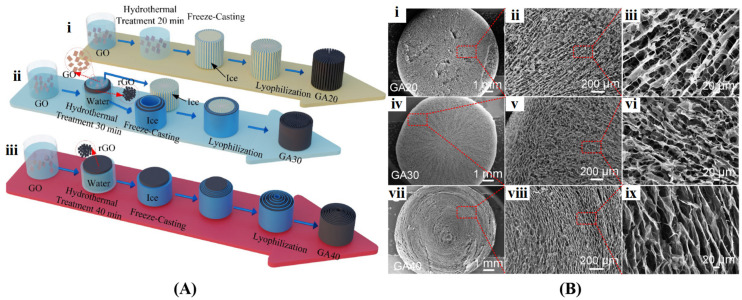
(**A**) The procedure of preparing graphene aerogels by a combination of hydrothermal treatment (different time), lyophilization and hydrazine reduction: 20 min (**i**), 30 min (**ii**), and 40 min (**iii**); (**B**) The cross-sectional SEM images of graphene aerogels with different hydrothermal times and their corresponding magnified views: 20 min (**i**–**iii**), 30 min (**iv**–**vi**), 40 min (**vii**–**ix**). Reproduced with permission from Ref. [97], Copyright 2024, Elsevier.

**Figure 8:**
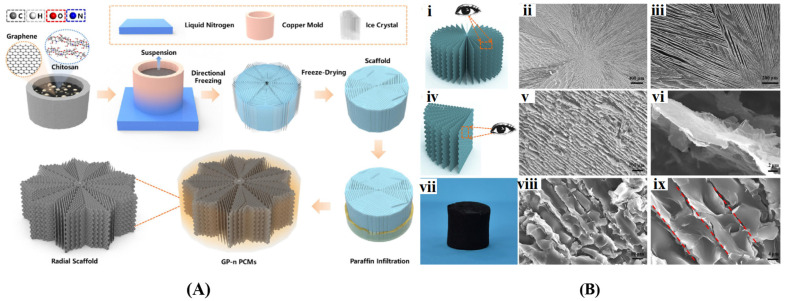
(**A**) Fabrication procedure of the phase change materials with radial scaffold; (**B**) Morphology of the aerogel and phase change material: schematic diagram for cross-sectional SEM observation (**i**) and cross-section SEM (**ii**,**iii**) of graphene/chitosan aerogel, schematic diagram for longitudinal section SEM observation (**iv**) and longitudinal section SEM (**v**,**vi**) of graphene/chitosan aerogel, digital image (**vii**) and SEM images of graphene/chitosan-PCM (**viii**,**ix**). Reproduced with permission from Ref. [98], Copyright 2024, Elsevier.

The ice template method is widely utilized for creating oriented microstructure materials with homogeneity due to its simplicity and ease of operation. This method relies on the segregation of the solidifying solvent template from the target phase through ice sublimation under extremely low temperature and vacuum conditions, resulting in porous materials with a templated pore structure. Common solvents used in ice templates include water, camphene, and tert-butanol, with water being the most frequently used dispersing medium due to its abundance, low cost, and non-toxic nature [96]. Zong et al. [97] introduced a bidirectional freeze-casting method to fabricate graphene aerogels with three distinct microstructures by employing a dual temperature gradient approach (Figure 7). They began with a graphene oxide aqueous solution prepared using the Hummers method, which was mixed with ascorbic acid and heated at 70 °C for varying durations to produce graphene hydrogels. Subsequently, these hydrogels were respectively immersed in −35 °C ethanol for 5 h, followed by thawing at room temperature. The final graphene aerogels were obtained after lyophilization and hydrazine reduction. The study demonstrated that the oriented microstructure of the graphene aerogels could be precisely controlled by adjusting the hydrothermal duration and the dual temperature gradient, which influenced ice crystal growth. Qiu et al. [98] fabricated 3D graphene nanoplates with vertical channels extending along the radius, driven by the growth of crystals in a radial pattern (Figure 8). They dispersed commercial graphene nanoplates into a solution of itaconic acid and chitosan using sonication. The mixture was then rapidly cryogenically frozen using liquid nitrogen at −60 °C for 30 min to promote the orientation of ice crystals. Subsequently, the frozen sample underwent freeze-drying to sublimate water and obtain the graphene/chitosan composite aerogel. Kota et al. [99] fabricated 3D nitrogen-doped graphene monoliths with a mesoporous structure using ice-templated assembly, incorporating melamine as the nitrogen source. After homogeneously blending the graphene oxide solution with melamine, the mixture was subjected to cryogenic freezing using liquid nitrogen, followed by thawing. The graphene oxide was then significantly reduced by annealing at 900 °C for 30 min, leading to the formation of a more robust porous structure.

#### 3.1.2. Soft Template Method

**Figure 9:**
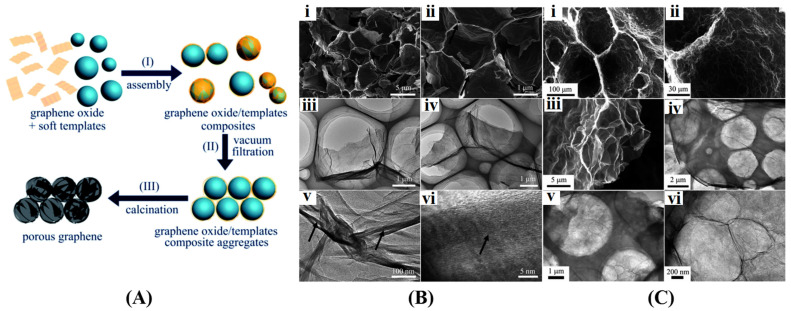
(**A**) Schematic illustration of the emulsion soft-template synthesis procedures for preparing porous graphene foams; (**B**) SEM images (**i**,**ii**) and TEM images (**iii**–**vi**) of graphene using TMB as emulsion templates; (**C**) SEM images (**i**,**ii**) and TEM images (**iii**–**vi**) of graphene using n-hexadecane as emulsion templates. Reproduced with permission from Ref. [101], Copyright 2014, Royal Society of Chemistry.

Soft templates, typically organic molecules that self-assemble into micelles or other nanoscale structures in a liquid phase, provide a simpler and milder alternative to hard templates for controlling the shape and growth of rigid particles [100]. Huang et al. [101] initially proposed a strategy for fabricating 3D graphene foams with tunable pore structures using microemulsions and micelles as soft templates (Figure 9A). The process began with the introduction of TMB (or n-hexadecane) into 2M HCl and sonication to create a cloudy suspension. This suspension was then combined with an aqueous graphene oxide solution, allowing the graphene oxide sheets to spontaneously arrange around the templates due to hydrophobic interactions. The resulting composites were separated via vacuum filtration and subjected to dual-step calcination under an inert atmosphere to obtain the final graphene material. This approach enabled the production of graphene with adjustable pore diameters ranging from a few micrometers to tens of micrometers, as illustrated in Figure 9B,C. Li et al. [102] employed a soft template-assisted approach combined with heat-pyrolysis to synthesize N and S co-doped 3D multi-porous graphene. Their method involved using melamine as a crosslinking agent and nitrogen source, and benzyl disulfide as the sulfur source. Initially, graphene oxide dispersion was mixed with melamine and formaldehyde aqueous solution to form a homogeneous mixture. Subsequently, the mixture was subjected to hydrothermal treatment at a temperature of 180 °C for 12 h to form a hybrid hydrogel, which was dried overnight at 80 °C to produce xerogel. Finally, the resulting xerogel underwent pyrolysis in an argon atmosphere to form N and S co-doped graphene.

### 3.2. Non-Template Method

#### 3.2.1. Self-Assembly Method

Self-assembly has been recognized as a highly effective strategy in the realm of ‘bottom-up’ nanotechnology. It is also a prevalent method for fabricating graphene and graphene-based composites featuring three-dimensional structures, capable of establishing intermolecular cross-links through hydrogen bonding, π-π stacking interactions, electrostatic forces, etc. [103]. This method offers advantages in terms of suitability for large-scale production due to its simple process. This section discusses the synthetic strategies for creating 3D graphene macrostructures from graphene oxide through self-assembly under hydro/solvothermal synchronous thermal reduction and chemical reduction processes.

##### Hydrothermal/Solvothermal Reduction

**Figure 10:**
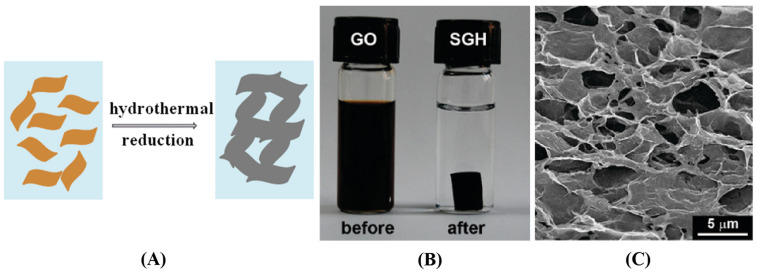
(**A**) The formation mechanism for graphene hydrogel; (**B**) Photographs of graphene oxide solution before and after hydrothermal reduction; (**C**) SEM image of the interior microstructures of graphene. Reproduced with permission from Ref. [104], Copyright 2010, American Chemical Society.

The hydrothermal method has become a leading approach for synthesizing interconnected 3D porous graphene hydrogels, notably advanced by Shi et al. [104]. Their work demonstrated that treating a homogeneous graphene oxide aqueous solution at 180 °C for 12 h yields a three-dimensional graphene hydrogel with a porous network. Graphene oxide’s hydrophilicity and electrostatic repulsion effect enable stable dispersion in water. Hydrothermal reduction then removes most oxygen-containing functional groups, enhancing the material’s hydrophobicity. This leads to the random stacking of flexible graphene sheets through π-π interactions, forming a porous framework with pore sizes ranging from sub-micrometer to several micrometers (Figure 10). Building on this foundation, they further prepared the 3D macrostructure graphene organogel using solvothermal reduction of graphene oxide dispersed in propylene carbonate [105]. Both hydrothermal and solvothermal reduction are the most direct methods for preparing graphene gels without requiring additional purification treatment. Moreover, these processes are compatible with synthesizing many functional materials, facilitating the incorporation of multiple active components into graphene frameworks, greatly expanding the diversity and functionality of the self-assembled graphene macrostructures. Numerous researchers have reported the construction of three-dimensional graphene/metal composites [106,107,108], graphene/metallic compounds [109,110,111,112], and graphene/polymers [113,114,115] hybrids through hydrothermal or solvothermal methodologies.

##### Chemical Reduction

In contrast to the high-temperature and high-pressure conditions required by the hydro/solvothermal method, the chemical reduction approach necessitates slightly milder experimental parameters. It is one of the conventional and economical methods for preparing graphene in large quantities through the reduction of graphene oxide. For chemical reduction, by altering the properties and mass of reducing agents, the surface chemistry can be readily adjusted to achieve the self-assembly of 3D graphene. Wang et al. [116] developed a method for the fabrication of macroscopic porous graphene aerogels using a mild in situ self-assembly process combined with thermal annealing. Sodium bisulfite (NaHSO_3_) was employed as the reducing agent in this approach. The chemical reduction took place at 80 °C under atmospheric pressure, which enhanced the surface hydrophobicity of graphene oxide. This change in hydrophobicity strengthened the π-π interactions between adjacent graphene sheets, promoting self-assembly into a 3D network. By varying the mass ratio of graphene oxide to NaHSO_3_ during similar processes and conditions, the self-assembly behavior and the resulting graphene aerogel’s density can be regulated. Higher reducing agent concentrations led to lower-density aerogels due to excessive accumulation of reduced graphene sheets. Chen et al. [117] carried out research on the effects of various reducing agents including NaHSO_3_, Na_2_S, Vitamin C, HI, and hydroquinone on the reduction of graphene oxide. Their study highlighted that different reducing agents influenced the reduction times required for forming 3D structures. Additionally, altering the reactor type also enabled precise manipulation of the shape of the hydrogel, revealing the isotropic contraction of self-assembled graphene oxide sheets. The flexibility of chemical reduction in manipulating reducing agents and reaction conditions offers a valuable strategy for tailoring the properties and structures of 3D graphene materials, rendering it a versatile approach for large-scale and economical graphene production.

#### 3.2.2. 3D Printing Method

**Figure 11:**
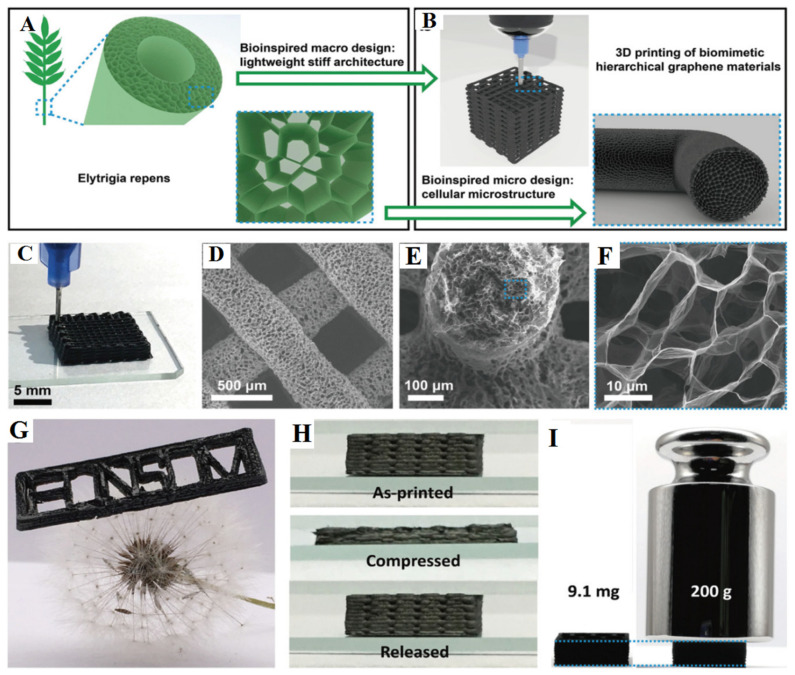
Schematic illustration of 3D printing (**A**–**C**); SEM images (**D**–**F**), ultralight structure (**G**), and mechanical properties (**H**,**I**) of graphene. Reproduced with permission from Ref. [132], Copyright 2019, Wiley.

3D printing is an additive manufacturing technique that builds objects layer by layer based on digital models [118]. This method is particularly promising when combined with hybrid graphene materials, as it allows for the rapid creation of complex 3D structures with specific performance and functional requirements. Currently, several 3D printing methods are used to manufacture graphene-reinforced composites, including inkjet printing [119,120], direct ink writing [121,122,123,124,125], fused deposition modeling [126,127,128], and the light-curing molding method [129,130,131]. Of these techniques, the direct ink writing method is the most versatile strategy for assembling satisfying three-dimensional hybrids due to its flexibility in material selection and facile printing process. Compared to conventional bulk graphene aerogels, the graphene materials obtained through 3D printing showcase unique micro- and macroscale architectures, resulting in exceptional mechanical properties (stiffness and stretchability) at a low density. For example, Peng et al. [132] employed an inkjet 3D printing methodology to fabricate ultralight graphene structures. This 3D printing process was to continuously squeeze the highly viscous paste onto a glass wafer at room temperature in the air by precisely controlling nozzle movement. They optimized partially reduced graphene oxide ink and combined it with freeze-casting and thremal reduction processes to achieve a hierarchical structure with both a macroscopically hollow scaffold and microscopically cellular features (Figure 11). Hu et al. [133] functionalized graphene oxide with amphiphilic polyethylene glycol to produce inks with stable dispersion properties in various organic and aqueous solutions for printing self-supporting 3D structures. Tang et al. [134] developed a versatile 3D printing approach for the manufacturing of graphene-based aerogels with complex architectures. In their printing process, urea was employed as a precursor to crosslink graphene oxide sheets and incorporate negatively charged materials (0D Ag nanoparticles, 1D multiwalled carbon nanotubes, and 2D MoS_2_ nanosheets) into the graphene oxide matrix. This approach allowed the creation of homogeneous ink systems and produced hybrid aerogels with intricate internal structures through 3D printing, followed by freeze-drying and chemical reduction.

## 4. Synthesis of 3D Graphene-Based Composite Photocatalysts

The conversion of solar energy into chemicals within photocatalytic systems relies on crucial processes: light absorption, charge separation, and surface reactions. Three-dimensional structured graphenes have emerged as highly effective materials for creating efficient photocatalysts due to their unique macrostructure and porous properties. 3D graphene enhances photocatalytic activity in multiple ways. Its outstanding electrical conductivity and extensive electron transport pathways make it an excellent mediator and acceptor for photoelectrons, thus promoting the effective separation of electron-hole pairs generated by light. Additionally, the large surface area and complex porous structures of 3D graphene improve adsorption capacity and prevent the aggregation of semiconductor particles. This structure not only provides more active sites for surface catalytic reactions but also supports better performance. Moreover, 3D graphene can act as a photosensitizer to intensify light harvesting and extend the light adsorption range of composite photocatalysts [135,136]. In this section, we review the synthesis strategies of various 3D graphene-based photocatalysts and explore how to fully use the diversiform roles of graphene in photocatalytic systems by adjusting active components and optimizing structure.

### 4.1. 3D Graphene/Metal Oxides

Metal oxides are currently the most widely employed photocatalysts due to their cost-effectiveness, chemical stability, as well as low toxicity. Of these, titanium (TiO_2_) stands as the most extensively studied, abundantly available, and eco-friendly n-type semiconductor. However, its relatively large band gap of about 3.2 eV restricts its light absorption to the ultraviolet region, and the rapid recombination of photogenerated charge carriers results in low quantum yield. Recently, numerous studies have been conducted to enhance photocatalytic activity through the integration of nano TiO_2_ with 3D graphene. Liu et al. [137] synthesized TiO_2_/graphene aerogels with uniformly distributed TiO_2_ nanorods using a hydrothermal and freeze-drying method for the photocatalytic reduction. The total yield of carbon generated by TiO_2_/graphene was 15.7 times higher than that of pure P25, which is attributed to the expansion of the light response range and the enhancement of interface charge transfer to mitigate electron-hole pair recombination. Huang et al. [138] employed chemical vapor deposition to synthesize high-quality graphene, which was then combined with TiO_2_ nanoparticles through the sol-gel method for photocatalytic hydrogen evolution. Benefiting from the low defect density and excellent electrical conductivity of few-layer graphene, the separation and lifetime of photoexcited charge carriers in the graphene/TiO_2_ photocatalyst were enhanced, resulting in high sustained stability with a hydrogen evolution rate 26.2 times higher than that of blank TiO_2_. Nawaz et al. [139] employed an in situ hydrothermal method to create TiO_2_/graphene aerogels for the photodegradation of recalcitrant carbamazepine. The composites exhibited enhanced adsorption and nearly doubled photodegradation capability compared to bare TiO_2_, attributed to the macroporous structure, effective charge separation, and efficient mass transportation of carbamazepine to the photocatalyst surface. Additionally, 3D graphene/TiO_2_ heterojunction photocatalysts have garnered significant attention for their ability to prolong charge carrier recombination times through built-in electric fields. Liu et al. [140] prepared TiO_2_/C/BiOBr graphene aerogel with an indirect Z-scheme heterojunction through the combination of electrostatic spinning and solvothermal methods. Many BiOBr nanosheets were grown on the surface of TiO_2_/C nanofibers to form a 3D hierarchical nanostructure in conjunction with graphene. The results of photocatalytic experiments revealed that the composite photocatalyst achieved a degradation rate of 97.5% for RhB, approximately 8.7 times higher than that of TiO_2_/C. This enhancement is attributed to the synergistic effect of the heterojunction formed between TiO_2_ and BiOBr as well as the improved conductivity and physisorption resulting from graphene. By optimizing the relative proportions of each constituent and the configuration of photocatalysts, Jung et al. [141] developed a composite material featuring mesoporous TiO_2_ and layered MoS_2_ cocatalyst on 3D graphene for CO_2_ photoreduction. The electron transfer from TiO_2_ through graphene to MoS_2_ effectively reduced the charge recombination rate, resulting in a higher CO photoconversion rate of 97% and a production yield of 92.33 μmol/g·h, outperforming other combinations such as bare TiO_2_, TiO_2_/graphene, and TiO_2_-MoS_2_ under simulated sunlight irradiation.

**Figure 12:**
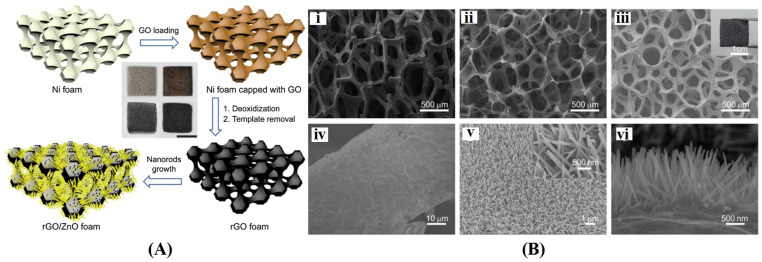
(**A**) Preparation schematic diagram of ZnO/graphene foam, inset is the corresponding sample photographs of each step; (**B**) SEM images of graphene/Ni (**i**), graphene (**ii**), and ZnO/graphene (**iii**–**vi**). Reproduced with permission from Ref. [142], Copyright 2016, Elsevier.

Many other metal oxides incorporating 3D graphene materials have also been prepared and applied in photocatalysis. Zinc oxide (ZnO) is an n-type wide gap semiconductor similar to TiO_2_, with high photosensitivity, relatively low cost, and environmental compatibility. Thus, ZnO has the same limitation as TiO_2_ in terms of rapid electron/hole recombination. Men et al. [142] created a 3D self-supporting graphene structure using Ni foam as a template, and subsequently grew ZnO nanorods on the graphene surface through a hydrothermal process, as illustrated in Figure 12. The ZnO/graphene composite foam features a micro-nano hierarchical structure; the microporous graphene framework not only enhances the light harvesting but also functions as an electron storage reservoir to facilitate the separation of electrons and holes, leading to the higher photocurrent and photocatalytic degradation activity of RhB compared to pure ZnO. Iron oxide (α-Fe_2_O_3_) is an earth-abundant, environmentally friendly n-type semiconductor with a relatively narrow band gap (∼2.1 eV), which makes it responsive to visible light (absorbance edge ∼600 nm). Despite these advantages, the utilization of α-Fe_2_O_3_ has been constrained by the high electron/hole recombination effect and low diffusion length [143]. Wang et al. [144] utilized a microgel template-assisted solvothermal method to synthesize uniform hollow α-Fe_2_O_3_ microspheres, and subsequently employed direct writing 3D printing to fabricate α-Fe_2_O_3_/graphene aerogel microreactors. The resulting macroscopic porous and highly interconnected networks exhibit a high specific surface area and structural stability, thereby enhancing multi-dimensional mass transfer channels and improving reusability. Additionally, the conducting graphene adjacent to the α-Fe_2_O_3_ facilitates rapid electron transport, leading to the efficient formation of photoinduced holes in the α-Fe_2_O_3_ microspheres. Under 120 min of solar irradiation, the degradation rate of RhB reached 97.8%, while maintaining 96.2% catalytic stability after four cycles. Tungsten trioxide (WO_3_) is another n-type semiconductor known for its non-toxicity, photochemical stability, and visible light absorption (up to ~480 nm) with a bandgap of 2.6–3.0 eV. Pure WO_3_ often suffers from low charge carrier mobility and rapid recombination of photogenerated carriers [145,146,147]. Enhancements in the performance of WO_3_ can be achieved through structural and morphological adjustments as well as the incorporation of high-quality conductive graphene. Azimirad et al. [148] prepared graphene foam through chemical vapor deposition and utilized it as a conductive substrate for the nucleation and growth of WO_3_ nanoparticles. The visible light photocatalytic activity of the 3D WO_3_/graphene composite in RhB degradation was found to be strengthened compared with pure WO_3_ due to the formation of W-C and W-O-C bonds between graphene and WO_3_, which facilitates faster photoexcited electron transfer to graphene. Li et al. [149] used a one-step hydrothermal method to synthesize 3D graphene-wrapped WO_3_ microspheres assembled with nanowires. The hierarchical structure of WO_3_ and the intimate wrapping of graphene on the nanowire surface synergistically improved the photocatalytic activity of WO_3_/graphene in degrading phenol and RhB. Additionally, the content of graphene played a crucial role in improving photocatalytic performance, with optimal performance reaching about 2.5 times that of bare WO_3_. Cuprous oxide (Cu_2_O) is a typical p-type semiconductor characterized by its low cost, non-toxicity, and bandgap of approximately 2.0 eV, enabling a specific response to visible light. However, its broad applicability is constrained by the rapid recombination of photogenerated carriers. Additionally, the stability of Cu_2_O poses a significant issue in redox reactions. The efficient coalescence between Cu_2_O and graphene not only enhances electron transport but also modulates the stability of the anchored Cu_2_O crystals [150,151,152,153].

### 4.2. 3D Graphene/Metal Sulfides

Compared to metal oxides, metal sulfides offer several advantages for photocatalysis. The valence band of metal sulfides, which is occupied by S 3p orbitals, is more negative than that of metal oxides, where the valence band is occupied by O 2p orbitals. This difference allows metal sulfides to harvest a broader range of light and achieve higher carrier concentrations. The versatility in selecting and adjusting various metal elements further enhances the electronic structure and surface physicochemical properties of metal sulfides, making them highly tunable for photocatalytic applications [154,155]. Thus, MoS_2_, CdS, WS_2_, CuS, and other binary metal sulfides with narrow bandgaps are widely recognized as efficacious visible light photocatalysts. Nevertheless, the photocatalytic efficiency of metal sulfides remains unsatisfactory primarily due to the sluggish kinetics of carrier separation and migration. And the relatively low chemical stability caused by the photo corrosion of surface sulfide ions severely impedes highly efficient solar energy conversion. Recent studies have shown that combining metal sulfides with graphene can significantly improve their optoelectronic properties [156]. For example, Das et al. [157] synthesized hierarchically porous and macroscale MoS_2_/graphene photocatalysts using a one-pot hydrothermal method. This approach assembled rosette-like MoS_2_ nanoflowers into the interconnected networks of graphene aerogels, resulting in composites with a narrow bandgap (~1.3 eV) and enhanced photon absorption across the visible light spectrum up to 700 nm. The extensive planar interface between MoS_2_ and graphene facilitates efficient spatial separation of photoinduced charge carriers, while the micro/mesoporosity of the structure improves interactions with reactants. The optimal MoS_2_/graphene aerogel demonstrated a photocatalytic degradation efficiency of 91% for tetracycline, about 1.7 times higher than that of pure MoS_2_, and retained its crystallinity and integrity after three repeated cycles. Wei et al. [158] used an in situ hydrothermal and freeze-drying method to assemble spherical CdS nanoparticles (~10 nm) on graphene aerogel. The resultant hybrids feature a hierarchical porous structure and a robust electronic interaction between CdS and graphene, thereby leading to the significantly enhanced adsorption capacity of reactants and efficient transfer of photo-generated carriers. This composite exhibited significantly improved photocatalytic degradation of organic contaminants, with efficiencies 15.6, 6.6, 4.4, 2.8, and 2.2 times higher for MO, MB, CIP, RhB, and AcbK, respectively, compared to pure CdS. Bano et al. [159] synthesized a CuS/graphene hybrid aerogel through a chemical reduction process to achieve the synergistic photocatalytic reduction of Cr (VI) and degradation of cationic dyes (MB and RhB). In comparison with powdered CuS nanospheres, this 3D porous framework facilitated better spatial separation and transport of photoinduced charge carriers, resulting in enhanced visible light catalytic activity and improved recyclability.

By leveraging the exceptional electron mobility of graphene as an electron trap to stimulate charge separation in semiconductors, the photocatalytic activity can be further augmented through doping with heteroatoms (such as N) to customize the electronic and structural properties of graphene [160]. For example, Shafi et al. [161] proposed a novel photocatalyst by in situ growing few-layer WS_2_ nanosheets on N-doped graphene aerogel through hydrothermal processing. The coexistence of pyridinic N species and WS_2_ clusters synergistically provides abundant active sites for catalytic reactions, while the interconnected conductive networks effectively regulate charge separation and migration. This combination allowed the WS_2_/graphene composite to achieve a degradation of up to 93% of the psychoactive drug caffeine within 180 min under visible light irradiation. Zhang et al. [162] employed a bottom-up approach to fabricate CoS_2_/MoS_2_/N-graphene aerogel photocatalysts. The heterostructure formed by CoS_2_ nanoparticles and ultra-thin MoS_2_ nanosheets connected via S-atoms chemical bonding can effectively prevent the aggregation of nanoscale MoS_2_, thereby exposing more edge active sites and accelerating charge separation at the heterojunction interface. Moreover, N-doped graphene served as the substrate for CoS_2_/MoS_2_ heterostructures to enhance adsorption, facilitate the migration of photogenerated electrons, and promote photocatalyst recovery. As indicated by the results, the composite exhibited a photocatalytic efficiency of 97.1% for RhB degradation, surpassing pure MoS_2_ (51.7%) by a significant margin.

### 4.3. Other 3D Graphene-Based Photocatalysts

In addition to metal oxides and metal sulfides semiconductor materials, graphitic carbon nitride, bismuth-based materials, and metal-organic frameworks are also considered as promising photocatalysts. Non-metallic g-C_3_N_4_ is a polymer semiconductor with a bandgap of 2.7 eV, structured as a 2D graphitic lattice formed by tri-s-triazine units linked with amino groups. It possesses several beneficial properties, including chemical stability, low cost, environmental friendliness, and facile preparation through the thermal polycondensation of diverse nitrogen-containing precursors [163]. However, g-C_3_N_4_ poses great challenges for practical applications due to the high recombination rate of photogenerated carriers, low electronic conductivity, and limited absorbance of visible light below 460 nm [164]. To address these issues, constructing heterojunctions between g-C_3_N_4_ and graphene can enhance photocatalytic performance by improving charge carrier separation and extending light absorption. To optimize its charge carrier separation and broaden the light absorption region, the construction of heterojunctions between g-C_3_N_4_ and graphene is anticipated to deliver excellent photocatalytic properties. Zhang et al. [165] prepared a porous g-C_3_N_4_/graphene aerogel photocatalyst using a hydrothermal co-assembly method. The formation of heterojunctions effectively mitigated electron-hole recombination and intensified visible light utilization through multireflection across the 3D interconnected porous frameworks. Furthermore, the large planar interface between g-C_3_N_4_ and graphene sheets increased the active sites, resulting in an 83.0% purification efficiency for dye MB within 3 h under visible light. To further suppress the high carrier recombination rate of g-C_3_N_4_, Zhang et al. [166] developed an efficient aerogel photocatalyst embedding palladium (Pd) within g-C_3_N_4_/graphene for the conversion of CO_2_ to CH_4_. In this photocatalytic system, Pd nanoparticles acted as electron traps to enhance electron-hole separation, while graphene provided a macroscopic substrate that facilitated contact CO_2_ and light energy utilization. The integration of Pd with g-C_3_N_4_ and graphene established a 2D-2D electron pathway, resulting in a maximum CH_4_ evolution rate of 6.4 μmol/g·h, a 12.8-fold increase compared to pure g-C_3_N_4_.

Bismuth-based semiconductors (such as Bi_2_O_3_, Bi_2_S_3_, BiVO_4_, BiOBr, Bi_2_WO_6_, etc.) are promising candidates for photocatalysis due to their unique electronic band structures, adjustable and expandable spectral response ranges from 400 nm to 700 nm, and low toxicity [167]. Despite these advantages, they suffer from rapid recombination of photogenerated carriers and high susceptibility to photo corrosion. Crystal facet engineering, ion doping, and coupling with noble metals or graphene materials are effective strategies to improve the characteristics of Bi-based photocatalysts [168]. Yu et al. [169] prepared a series of BiOBr/graphene aerogels using a two-step hydrothermal method, where flower-like BiOBr self-assembled on the graphene surface to form heterostructures with dopamine as a cross-linker. An increase in redshift and absorption intensity in the visible light range was observed with the increasing graphene content. The optimal ratio of BiOBr/graphene showed higher selective adsorption and degradation of anionic MO compared to BiOBr because of strong π-π interactions with dye through the conjugate aromatic structure. Furthermore, these aerogels serving as bulk catalysts can be easily retrieved from photocatalytic systems for recycling. Yang et al. [170] fabricated a composite aerogel by means of a two-step hydrothermal procedure, combining BiVO_4_ quantum tubes as the light-absorbing constituent with graphene as a structural support and photoelectron transport pathway. The improved interfacial charge carrier transfer leads to more efficient spatial charge separation. The BiVO_4_/graphene composite photocatalyst exhibited favorable catalytic performance, capable of degrading gaseous formaldehyde from 1.0 ppm to 0.4 ppm within 15 min, approximately three times faster than pristine BiVO_4_.

Metal-organic frameworks (MOFs) are crystalline materials characterized by their periodic network structure units, composed of metal ions/metal clusters and organic ligands connected through coordination covalent bonds. MOFs possess the advantages of abundant catalytic active sites, diverse pore structures, high surface area, and distinctive optical properties, rendering them an extremely appealing material in photocatalysis [171,172,173,174]. However, pristine MOFs still encounter challenges such as limited stability, weak conductivity, and inefficient separation of electron-hole pairs. To address these issues, integrating MOFs with graphene has proven to be an effective strategy. The hydrophobic nature of graphene enhances the stability of MOFs in aqueous environments, while π-π stacking interactions between graphene sheets and MOFs improve their overall performance [175]. For example, Zhao et al. [176] synthesized a series of DUT-67 (Zr-MOF)/graphene photocatalysts with 3D morphology through hydrothermal processing, enabling the selective catalytic conversion of CO_2_ by harnessing the synergistic effect between the macrostructure of graphene and DUT-67. The incorporation of graphene and aerogel structure enhanced the photo charge separation efficiency of DUT-67, thus promoting more charges to participate in the photocatalytic reaction. The optimized ratio of DUT-67/graphene composites achieved near 99.6% selectivity in converting CO_2_ to CO. Similarly, Shah et al. [177] developed a hybrid photocatalyst by linking 3D graphene with NH_2_-MIL-125 (Ti-MOF) through a solvothermal method with NH_2_-IL serving as bridging. The designed hybrid photocatalyst exhibited a higher degradation efficiency (97%) for acetaldehyde compared to pure NH_2_-MIL-125 (57%), attributed to the intimate interfacial contact, high dispersion, excellent surface area, and improved electrical conductivity.

## 5. Applications of 3D Graphene-Based Photocatalysts

In the preceding sections, we explored the structural advantages of three-dimensional graphene and various synthesis methods, along with the preparation and photocatalytic performance of graphene-based photocatalysts. This section will focus on their applications in environmental photocatalysis, specifically their roles in oxidizing organic pollutants, hydrogen evolution, and CO_2_ reduction. A summary of the photocatalytic activity and reaction conditions of these systems is presented in Table 2. Due to space limitations, this section will concentrate on the impact of 3D graphene on photocatalytic reactions.

### 5.1. Photocatalytic Degradation of Organic Pollutants

**Figure 13:**
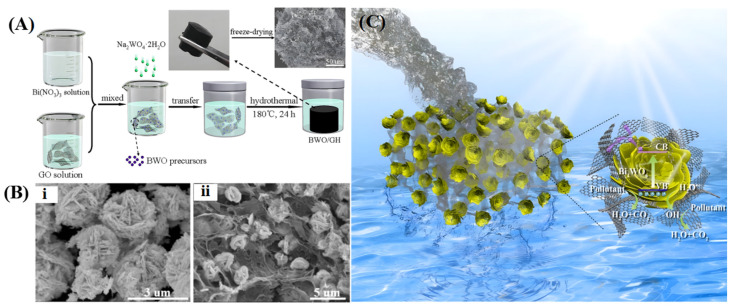
(**A**) Schematic illustration of the synthetic process of Bi_2_WO_6_/graphene; (**B**) SEM images of Bi_2_WO_6_ (**i**) and Bi_2_WO_6_/graphene (**ii**); (**C**) Schematic diagram of pollutants adsorption and photocatalytic degradation by Bi_2_WO_6_/graphene composite. Reproduced with permission from Ref. [178], Copyright 2017, Elsevier.

**Figure 14:**
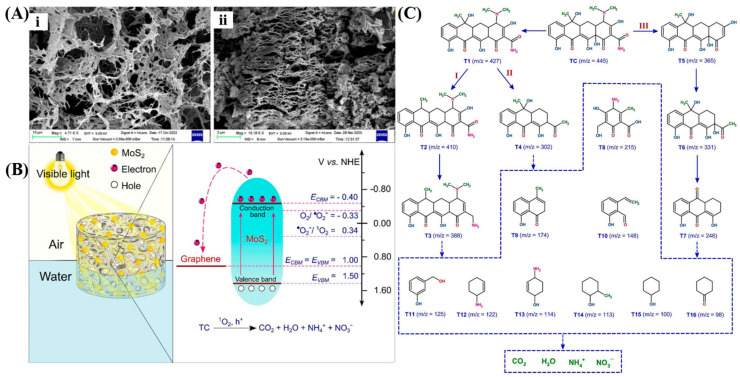
(**A**) SEM images of MoS_2_/graphene with different synthetic process: hydrothermal method (**i**) and chemical activation route (**ii**); (**B**) Schematic of the photocatalysis mechanism under visible−light irradiation; (**C**) Probable pathways for photocatalytic degradation of tetracycline. Reproduced with permission from Ref. [110], Copyright 2024, American Chemical Society.

The removal of contaminants from water bodies is crucial due to their detrimental effects on aquatic ecosystems and potential risks to human health. Photocatalysis stands out as a promising approach for wastewater treatment, capable of the degradation of various toxic and organic pollutants. In general, understanding the mechanism of photocatalytic reactions is key to designing effective semiconductor photocatalysts by predicting charge carrier pathways. Yang et al. [178] synthesized a composite photocatalyst by integrating flower-like Bi_2_WO_6_ with 3D porous graphene through a hydrothermal approach for the degradation of MB and 2,4-dichlorophenol, as shown in Figure 13. In this photocatalytic degradation process, graphene acted as a supporter for Bi_2_WO_6_, enabling rapid adsorption and enrichment of pollutants onto the composite’s surface. Upon exposure to visible light, Bi_2_WO_6_ was excited to generate electrons and holes. The holes in the valence band partially reacted with H_2_O to generate •OH radicals, both of which possess strong oxidation capabilities, resulting in the complete oxidation of pollutants into H_2_O and CO_2_. Furthermore, electrons in the conduction band of Bi_2_WO_6_ efficiently transferred to the 3D structural graphene due to its excellent conductivity, significantly improving electron-hole separation efficiency and enhancing overall photocatalytic performance. Jin et al. [179] prepared TiO_2_ nanowires/graphene 3D framework material for the photocatalytic degradation of micro-organic contaminant ethenzamide. The interconnected structure provides convenient migration pathways and abundant pores to enhance reactant adsorption and capture light through the refraction-reflection effect. Furthermore, the superior electron transport capability of graphene and the chemical bonding between TiO_2_ facilitate the rapid transition of photoelectrons, resulting in more e^−^ reacting with dissolved oxygen to form •O_2_^−^. Free radical trapping experiments confirmed that h^+^, •O_2_^−^, and •OH all contributed to the photocatalytic degradation process. Dong et al. [180] fabricated a macroscopic monolithic ZnSnO_3_/graphene aerogel for the adsorption and visible light photocatalytic degradation of ciprofloxacin wastewater. The incorporation of 3D graphene optimized the interfacial and electronic band structure, mitigating photogenerated electron-hole pair recombination and generating more •O_2_^−^ and •OH active species. Liu et al. [181] reported the fabrication of a Z-scheme-type CeVO_4_/3D graphene/BiVO_4_ ternary photocatalyst for visible-light-driven tetracycline degradation. Graphene plays a crucial role as an effective solid-state electron mediator in promoting electron-hole separation and improving light utilization. Das et al. [110] prepared 3D composites comprising MoS_2_ nanoflakes grown on graphene sheets via hydrothermal processing, followed by chemical activation with KOH solution under a high-temperature atmosphere to achieve more orderly mesopores and high-quality graphene layers for the photocatalytic degradation of tetracycline, as shown in Figure 14. The induction of mesoporosity augments light harvesting by facilitating both light penetration and scattering throughout the entire volume. In this photocatalytic system, the electrons photogenerated by MoS_2_ can be transferred to graphene on account of their work function differences, the interconnected graphene networks are capable of effectively inhibiting the recombination of electron/hole pairs by providing multidimensional transport channels. Reactive oxygen species scavenging experiments revealed that ^1^O_2_ and h^+^ primarily drive tetracycline dissociation, whereas •O_2_^−^ and •OH play an auxiliary role in the photocatalytic process. Additionally, the degradation pathways are hypothesized based on intermediate compounds identification, with all these intermediates eventually mineralizing to CO_2_, H_2_O, NH_4_^+^, and NO_3_^−^.

### 5.2. Photocatalytic Hydrogen Evolution

**Figure 15:**
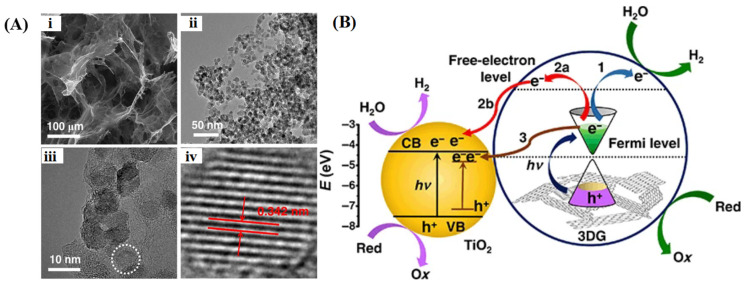
(**A**) A SEM image of 3D graphene (**i**), TEM images of TiO_2_/graphene (**ii**,**iv**), and the lattice diffraction pattern of the particle in the white circle in (**iii**); (**B**) Electron transfer pathways in the photocatalytic hydrogen production by TiO_2_/graphene. Reproduced with permission from Ref. [66], Copyright 2017, Springer.

**Figure 16:**
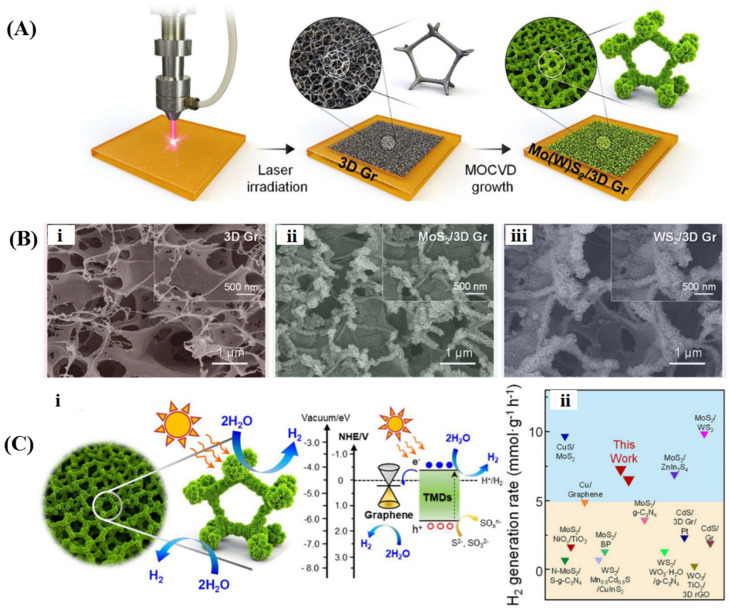
(**A**) Fabrication process of (MoS_2_ and WS_2_) nanosheets/3D graphene; (**B**) SEM images of 3D graphene (**i**), MoS_2_/graphene (**ii**), and WS_2_/graphene (**iii**); (**C**) Schematic illustration of the transfer process in the photocatalyst system (**i**) and the photocatalytic performance comparison of this work with other photocatalysts in the literature (**ii**). Reproduced with permission from Ref. [187], Copyright 2024, American Chemical Society.

The conversion of abundant solar energy into chemical energy through photocatalysis has garnered significant attention in recent years. Specifically, water photolysis for hydrogen evolution is considered an effective strategy to alleviate energy and environmental issues. To satisfy the thermodynamic prerequisites for redox reactions, an active photocatalyst must possess an appropriate bandgap and more negative conduction band edge than the reduction potential of H^+^/H_2_, as well as a more positive valence band edge than the oxidation potential of H_2_O to O_2_ [182,183]. The development of robust photocatalysts to improve the H_2_ production rate is a recent research hotspot. Lu et al. [66] combined the widely used semiconductor TiO_2_ with 3D graphene to fabricate a composite photocatalyst for efficient hydrogen production, where TiO_2_ particles (~10 nm) are uniformly distributed over the surfaces of graphene sheets (Figure 15A). Multiple pathways for hydrogen evolution may exist in the photocatalytic system (Figure 15B): graphene can act as an independent photocatalyst, utilizing electrons generated under light to reduce water to hydrogen (path 1); photogenerated electrons may transfer between graphene and TiO_2_, boosting photocatalytic activity (paths 2a and 2b); or graphene can reduce the TiO_2_ bandgap, thus promoting electron transfer from the Fermi level of graphene to the conduction band of TiO_2_ (path 3) and suppressing the recombination of charge carriers. Samajdar et al. [184] fabricated a Na_0.5_Bi_0.5_TiO_3_/graphene heterogeneous photocatalyst with a 2D/3D interface for catalytic hydrogen evolution under visible light irradiation. In this composite, graphene exhibits higher conduction band and valence band potentials than Na_0.5_Bi_0.5_TiO_3_, resulting in the formation of a Z-scheme heterojunction at the interface when they are in close contact. The effective separation of photogenerated charge carriers and enhanced electron reduction ability led to a significantly increased rate of H_2_ generation. Zhang et al. [185] developed a composite graphene hydrogel incorporating CdS nanoparticles. The interfacial contact and energy level matching between them favor the directional transfer of photoelectrons from CdS to graphene for hydrogen generation. The unique 3D network structure of graphene not only exposes more active sites for catalysis but also suppresses photo corrosion and enhances the charge migration of CdS nanoparticles. Liu et al. [186] synthesized a ternary CdS/g-C_3_N_4_/graphene aerogel to enhance the photocatalytic H_2_ production activity. CdS nanoparticles and g-C_3_N_4_ sheets were uniformly distributed within the 3D hierarchical networks of graphene, creating an S-scheme heterojunction to broaden the optical absorption range and promote the separation of photogenerated carriers. Seo et al. [187] constructed edge-rich 3D structured metal chalcogenide/graphene for photocatalytic hydrogen generation by combining laser irradiation with metal-organic chemical vapor deposition for the growth of MoS_2_ and WS_2_ nanosheets on porous graphene, as depicted in Figure 16. In this photocatalytic system, photogenerated electrons partake in the hydrogen evolution reaction at specified active sites, while holes are seized by sacrificial agents. The high electrical conductivity of graphene operates synergistically with the heterojunction effect between Mo(W)S_2_ and graphene to facilitate photocarriers participation in the catalytic reaction. Moreover, the configuration of graphene improved the density of accessible active sites, thereby escalating photocatalytic performance. The hydrogen generation rate of MoS_2_/graphene and WS_2_/graphene was comparable to that of previously reported composite materials.

### 5.3. Photocatalytic CO_2_ Reduction

**Figure 17:**
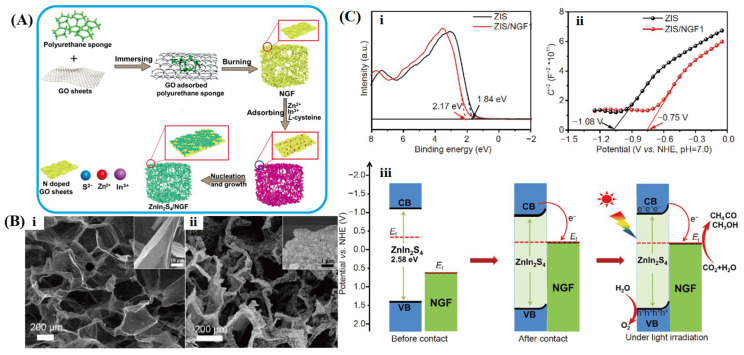
(**A**) Schematic illustration for the formation process of ZnIn_2_S_4_/N-graphene; (**B**) SEM images of N-graphene (**i**) and ZnIn_2_S_4_/N-graphene (**ii**); (**C**) Photogenerated charge transfer mechanism: valence band spectra (**i**), flat-band potentials (**ii**), and energy level diagram (**iii**). Reproduced with permission from Ref. [197], Copyright 2020, Springer.

In recent decades, the excessive consumption of fossil fuels has led to a persistent rise in atmospheric CO_2_, resulting in global warming and posing substantial ecological and environmental challenges. Against the background of carbon neutrality, developing efficient CO_2_ capture strategies and converting them into high-value-added chemical products has become one of the most environmentally friendly and sustainable approaches [188,189]. Since the pioneering work of Inoue et al. [190] in 1979 on the photocatalytic reduction of CO_2_ using semiconductors, significant progress has been made in the study of CO_2_ conversion through photocatalysis. Nevertheless, the practical application of photocatalytic CO_2_ reduction is greatly constrained by factors such as absorption capability, light utilization efficiency, electron-hole recombination rate, etc. Especially, the activation of CO_2_ is exceedingly challenging due to the high dissociation energy of the C=O bond in its molecule [191,192,193]. Regarding this matter, a further enhancement of CO_2_ reduction efficiency catalyzed by graphene-based photocatalysts has been proposed. For example, Park et al. [194] reported the synthesis of a nanocomposite photocatalyst through the covalent attachment of TiO_2_ nanoparticles to pristine 3D graphene. The TiO_2_/graphene exhibited enhanced photocatalytic activity for CO_2_ reduction to CO compared to bare TiO_2_, which can be attributed to the high specific surface area, strong interactions, and excellent dispersion of TiO_2_ nanoparticles on graphene. Zhang et al. [195] promoted the activation and conversion of CO_2_ by in situ growing ZnO nanowire arrays on the surface of 3D N-doped graphene. The efficiency of ZnO/N-graphene photocatalytic CO_2_ reduction to CH_3_OH was increased by 2.3 times. In this composite, N-doped graphene not only facilitated the uniform growth of ZnO but also improved the separation of electron-hole pairs and functioned as active sites for the adsorption and reduction of CO_2_. Song et al. [196] developed MOF-808/graphene aerogel materials for the photocatalytic reduction of CO_2_ to CO. The improved photothermal and photoelectric conversion efficiencies were associated with the three-dimensional macroscopic structure, which boasted a large specific surface area, abundant internal pore structure, and increased active sites. These factors collectively contribute to the enhancement of light energy utilization and the acceleration of the electron transfer rate. Xia et al. [197] reported a hierarchical composite photocatalyst for solar-driven CO_2_ reduction, created by assembling vertically aligned ZnIn_2_S_4_ nanowall arrays on N-doped graphene foam via hydrothermal synthesis, as illustrated in Figure 17A. This composite maintained the integrity of the 3D network structure while anchoring the ZnIn_2_S_4_ nanowalls securely onto the N-doped graphene surface (Figure 17B). ISI-XPS combined with flat-band potential measurements was employed to elucidate the energy band structure and photocatalytic mechanism (Figure 17C). Analysis using ISI-XPS and flat-band potential measurements revealed that electrons transfer from ZnIn_2_S_4_ to N-doped graphene when their Fermi levels align. Under simulated sunlight irradiation, photoelectrons migrate from ZnIn_2_S_4_ to N-doped graphene through the heterojunction interface. The N dopants possess a relatively strong electron affinity, functioning as polar sites and electron collectors to attract CO_2_ molecules and facilitate the reduction reaction of CO_2_ with photoelectrons on the graphene surface.

**Table 2:** Summary of photocatalytic applications over some 3D graphene-based photocatalysts.

Photocatalyst	Light Source	Reaction Type	Reaction Conditions	Performance	Ref
Bi_2_WO_6_/graphene	Xenon lamp (500 W) >420 nm	Degradation	40 ppm MB, 50 mL 5 ppm 2,4-CDP, 50 mL	Static system: 50.6% Dynamic system: 28.1% (MB), 17% (2,4-CDP)	[178]
TiO_2_/graphene	UV lamp (24 W) VUV lamp (1.2 W)	Degradation	500 ppb ethenzamide, 150 mL	98.5%, 60 min 99.8%, 3 min	[179]
ZnSnO_3_/graphene	Xenon lamp	Degradation	100 mg/L CIP, 100 mL	almost 100%, 120 min	[180]
CeVO_4_/graphene/BiVO_4_	Xenon lamp (500 W)	Degradation	20 mg/L TC, 100 mL	100%, 60 min	[181]
MoS_2_/graphene	Mercury lamp (250 W, with UV cutoff filter)	Degradation	5 mg/L TC, 100 mL	97%, 120 min	[110]
TiO_2_/graphene	Xenon lamp (200 W) 320~780 nm	H_2_ evolution	methanol (10 vol%)	1205 μmol·h^−1^·g^−1^	[66]
Na_0.5_Bi_0.5_TiO_3_/graphene	Xenon lamp (250 W)	H_2_ evolution	methanol (25 vol%)	100 mmol·h^−1^·g^−1^	[184]
CdS/graphene	Xenon lamp (500 W) 320~780 nm	H_2_ evolution	0.1 M Na_2_S·9H_2_O, 0.5 M Na_2_SO_3_	213.358 μmol·h^−1^·g^−1^	[185]
CdS/g-C_3_N_4_/graphene	Xenon lamp (300 W) >420 nm	H_2_ evolution	TEOA (5 vol%)	86.38 μmol·h^−1^·g^−1^	[186]
MoS_2_/graphene WS_2_/graphene	Xenon lamp (150 W)	H_2_ evolution	0.35 M Na_2_S, 0.25 M Na_2_SO_3_	6.51 mmol·h^−1^·g^−1^ 7.26 mmol·h^−1^·g^−1^	[187]
TiO_2_/graphene	Mercury lamp (200 W)	CO_2_ reduction	CO_2,_ triethylamine vapor	CO conversion: 1.26 μmol·mg^−1^	[194]
ZnO/N-graphene	Xenon lamp (300 W)	CO_2_ reduction	84 mg NaHCO_3_, 2M H_2_SO_4_	CH_3_OH conversion: 1.51 μmol·h^−1^·g^−1^	[195]
MOF-808/graphene	Xenon lamp	CO_2_ reduction	CO_2_, H_2_O	CO conversion: 14.35 μmol·g^−1^	[196]
ZnIn_2_S_4_/N-graphene	Xenon lamp (300 W)	CO_2_ reduction	84 mg NaHCO_3_, 2M H_2_SO_4_	CH_4_: 1.01 μmol·h^−1^·g^−1^ CO: 2.45 μmol·h^−1^·g^−1^ CH_3_OH: 1.37 μmol·h^−1^·g^−1^	[197]

## 6. Summary and Perspectives

Over the past decade, extensive research efforts have been dedicated to the systematic investigation of three-dimensional graphene materials owing to their distinctive structural and morphological characteristics. Incorporating photocatalytic active components into the graphene networks has created new possibilities for synergistically improving the performance of various photocatalytic processes. In this review, we commence by outlining the primary synthetic strategies for 3D graphene and graphene-based architectures, encompassing hard template methods (CVD, microsphere, and ice template methods), soft template methods, and non-template methods (self-assembly, chemical reduction, and 3D printing). It is evident that the selection of appropriate synthesis methods and optimization conditions is crucial for customizing the macroscopic structure, porosity, micro-morphology, defects, and surface/interface properties of 3D materials. Furthermore, recent advancements in the synthesis of 3D graphene-based composite photocatalysts are summarized herein; these include metal oxides, metal sulfides, g-C_3_N_4_, bismuth-based compounds, and MOFs. And we also attempt to comprehensively understand the multifaceted roles of 3D graphene in enhancing photoredox performance as well as the key factors influencing their catalytic activity. Finally, we elaborate on the applications of 3D graphene-based photocatalysts in organic pollutant degradation, hydrogen evolution, and CO_2_ reduction.

Despite the ongoing advancements in the design and synthesis of 3D graphene-based photocatalysts and understanding their reaction mechanisms, several challenges remain that require further attention. Firstly, with regard to practical applications, there is a pressing need to explore and optimize innovative approaches for the large-scale synthesis of highly active yet cost-effective photocatalysts in future research. Substantial efforts are also necessary for reactor design, stability, and the recyclability of photocatalysts to enable repeated use. Additionally, synergies between the photoactive components and 3D graphene skeleton are commonly cited to elucidate the enhanced photocatalytic properties. With the rapid advancement in experimental techniques, approaches such as in situ characterization and theoretical simulation can provide valuable insights into the growth mechanisms of photoactive materials on graphene. These approaches can also shed light on charge transfer dynamics and the real-time transformation pathways of reactant molecules and intermediates.

The brief history of 3D graphene-based photocatalysis showcases impressive progress, and we sincerely hope that this review may give some inspiration for further research in this field. From the initial laboratory investigations to practical application, the development of novel photocatalysts based on 3D graphene will be a lengthy process requiring constant efforts and substantial breakthroughs. However, it is certain that the integration of multidisciplinary knowledge will continue to open up the potential applications of 3D graphene-based photocatalysts in environmental and energy-related fields in the near future.

## Data Availability

Not applicable.
